# 21-Year-Old Female with Pneumothorax and Massive Air Leak Following Blunt Trauma; a Photo Quiz 

**DOI:** 10.22037/aaem.v10i1.1513

**Published:** 2022-04-09

**Authors:** Ahmad Shirinzadeh-Dastgiri, Ali Saberi, Mohammad Vakili, Sayed Mahdi Marashi

**Affiliations:** 1 Surgery Department, Iran University of Medical Sciences, Tehran, Iran.; 2 Surgery Department, Ardabil University of Medical Sciences, Ardabil, Iran.; 3 Patient Safety Research Center, Department of Forensic Medicine, School of Medicine, Iran University of Medical Sciences, Tehran, Iran.

**Keywords:** Echinococcus granulosus, Echinococcosis, Pulmonary, Lung Diseases, Parasitic, Pneumothorax

## 1. Case presentation

**Figure 1:**
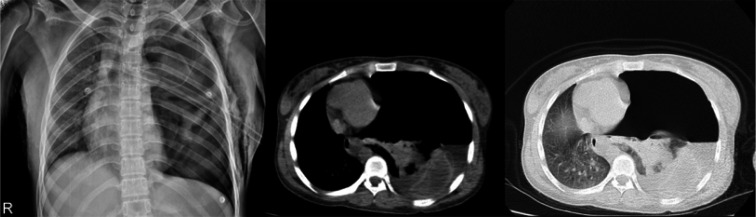
Posterior-anterior chest X-ray (left) and axial computed tomography (CT) scan in the mediastinal and lung window (middle and right) of patient

A 21-year-old female presented to the emergency department, about 20 minutes after a motorcycle accident. She was agitated and complaining of **shortness of breath**. Her vital signs were: heart rate 110/minute, respiratory rate 32/minute, blood pressure 89/67 mmHg, temperature 36.5°C, Oxygen saturation was 79% in room air, and GCS=15/15. Chest auscultation revealed decreased breath sounds on the left side. Other findings were tracheal deviation to the right side, distension of neck veins, decreased chest expansion and increased percussion note. Therefore, the diagnosis of pneumothorax was made for the patient and a left thoracostomy tube was inserted and about 200cc of serosanguineous fluid was drained. However, massive air leak was noted and the clinical symptoms did not improve. The patient underwent chest X-ray and computed tomography (CT) scan of the chest (Figure 1).

## 2. Diagnosis

**Figure 2:**
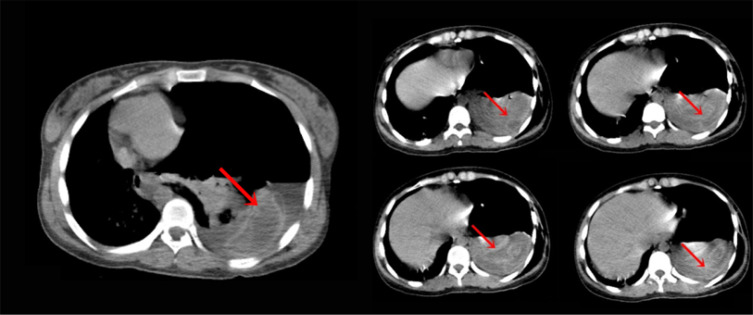
Axial computed tomography (CT) scan in the mediastinal window of patient, showing crumpled laminated membrane (red arrows).

The chest x-ray showed huge pneumothorax on the left side, severe contralateral shift of the mediastinum and trachea to the right, and collapsed left **lung** displaced rightwards (Figure 1, left); axial chest CT scan in the mediastinal window showed crumpled laminated membrane, suggestive for collapsed membrane of ruptured hydatid cyst following complete detachment into the pleural cavity (Figure 2; serpent sign; red arrows).

## 3. Case fate

**Figure 3:**
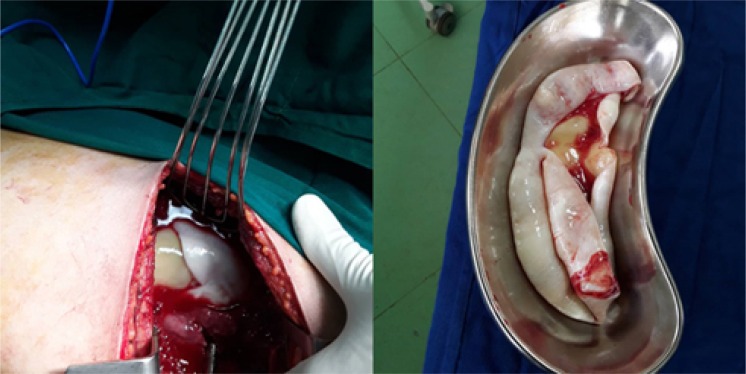
Intra-operative images of complete detachment of hydatid cyst into the pleural cavity

Laboratory tests revealed white blood cell count of 10800/μL, haemoglobin of 11 g/dL, and platelet count of 318000/μL. No further laboratory tests, including **Enzyme**-linked immunosorbent assay (**ELISA**) for the detection of **Echinococcus** granulosus, were requested due to the patient's emergency status. An emergency thoracotomy was performed, which showed complete detachment of hydatid cyst into the pleural cavity (figure 3). After removing the cyst, washing of the pleural cavity with isotonic saline was performed, fibrotic lung tissue was removed, *capitonnage was done, and a thoracostomy tube was inserted. The patient was discharged on day 5 of hospital admission with albendazole 800 mg/day for 3 months. The patient was doing well on follow-up 4 months later. Her physical examinations were normal. The patient was recommended for chest x-ray, but she refused.* 

## 4. Discussion:

Hydatic disease is a zoonosis disease caused by *Echinococcus* genus of tapeworm. The primary and intermediate hosts are carnivores and herbivores, respectively. Humans are accidentally infected after ingestion of comestibles contaminated with ova of the parasite (1). Cyst consists of the outer pericyst, the middle-laminated membrane, and the inner germinal layer, where the larval stage of the parasite is spent (2). The most common site of infection in adolescence and childhood are the liver and lungs, respectively (3, 4). 

Patients with** pulmonary hydatid cyst usually** present with nonspecific symptoms including chest pain, cough, dyspnea, or fever. **Cyst rupture** can occur spontaneously or after chest trauma. **Cyst** rupture **into the tracheobronchial tree** may cause** hypersensitivity reactions,** varying from **fever** or rash to** life-threatening anaphylaxis.** It may also become complicated by **pneumothorax (simple or tension**), bronchopleural fistula, **pulmonary collapse, or pleural empyema. Tension pneumothorax is a relatively rare condition**, with a prevalence of 1.3% (1, 4). The main **treatment of a ruptured pulmonary hydatid cyst** is surgery. The goal of surgery is to remove all the cysts, to prevent recurrence. The most **appropriate surgical procedure is** the one in which the **cyst membrane is removed**, bronchial openings are closed, and *capitonnage is performed.* Decortication, segmentectomy, or lobectomy **may** be required in** complicated cases. ** Extensive washing **with hypertonic saline** may prevent pleural hydatidosis during surgery.** Albendazole is given as a complementary treatment after surgery (**4**).**

The classical imaging findings of hydatid cyst are well-described and generally, they are incidentally found in r*adiologic* investigations; hence, imaging plays an important role in the diagnosis of complications. In cases of completely ruptured pulmonary hydatid cysts, connection with the bronchus signs, such as waterlily sign, rising sun sign, cumbo sign, dry cyst sign, and serpent sign may be seen (5). After perforation of the endocyst and expectoration of its internal fluid, membranes will collapse within the cyst and present with serpent sign (5).

## 5. Conclusion:

*R*uptured hydatid cyst has to be considered as a possible cause of pneumothorax with an associated pleural effusion in patients coming from endemic areas. 

## 6. Declarations:

### 5.1. Acknowledgements

None.

### 5.2. Conflict of interest

None.

### 5.3. Funding and support

None.

### 5.4. Authors’ contributions

 The authors meet the four criteria for authorship based on the recommendations of the International Committee of Medical Journal Editors (ICMJE).
